# Evaluation of the effect of home bleaching agents on surface microhardness of different glass-ionomer cements containing hydroxyapatite

**DOI:** 10.4317/jced.53852

**Published:** 2017-09-01

**Authors:** Farahnaz Sharafeddin, Mahsa Kowkabi, Soodabe Shoale

**Affiliations:** 1 Professor of Dept. of Operative Dentistry, Biomaterial Research Center, School of Dentistry, Shiraz University of Medical Sciences, Shiraz, Iran; 2 Postgraduate Student, Dept. of Operative Dentistry, School of Dentistry, Shiraz University of Medical Sciences, Shiraz, Iran

## Abstract

**Background:**

Home bleaching agents may exert some negative effects on surface hardness of restorative materials such as glass-ionomer cements (GICs). Since some studies have shown that some components such as hydroxyapatite (HA), as a bioactive glass, can improve the mechanical properties of dental materials, the effect of bleaching agents on surface hardness of GICs containing hydroxyapatite is questionable. This study was designed to evaluate the effect of home bleaching agents on the surface hardness of two different commercially available GICs containing hydroxyapatite.

**Material and Methods:**

80 disk-shaped specimens were made from two different GICs, including resin modified glass-ionomer and Zirconomer. Each material was divided into four groups (n=10): 1. control, 2. 20 %wt. hydroxyapatite-containing, 3. bleached and 4. bleached 20 %wt. hydroxyapatite-containing. Group 1 and 2 specimens were stored in distilled water for 2 weeks while group 3 and 4 specimens were treated with 15% carbamide peroxide in that period. Surface hardness was tested with Vickers surface hardness tester. Data were analyzed with 3-way ANOVA and mean comparison done by post hoc Tukey tests (*p*<0.05).

**Results:**

In general RMGI had a significantly highest Vickers surface hardness value among all groups. 15% carbamide peroxide reduced surface hardness compared to control groups (RMGI and Zr) significantly. In the HA-containing GICs groups, bleaching agent did not significantly changed the surface hardness value.

**Conclusions:**

In this study we concluded that applied treatments (bleaching and adding HA) in implicit percentages reduced surface hardness of GICs. Also we suggest more studies in clinical conditions be done to verify these results.

** Key words:**Home bleaching, Resin Modified Glass-ionomer cement, surface hardness, Zirconia-reinforced glass ionomer, hydroxyapatite.

## Introduction

Widespread use of bleaching agents for improving the aesthetic appearance of natural teeth began in 1990 after the introduction of home bleaching technique by Haywood and Heymann (1). As a result of the increasing demand for aesthetic procedures, many tooth-colored restorative materials have been introduced. One of these tooth-colored restorative materials is glass-ionomer cements (GICs) which have some advantages such as direct adhesion to the tooth structure (2), fluoride release (3), low microleakage and cytotoxicity (4); however, low strength and toughness are their disadvantages (5). Dental practitioners are seeking ways to overcome these disadvantages. Some researchers have recommend inserting components like hydroxyapatite (HA), glass and polyethylene fibers, and carbon and metal ingredients into glass-ionomer cements to overcome these drawbacks (6-8). HA is the main component of tooth and bone (9). The HA particles were added to glass-ionomer powder due to their biocompatibility and similar composition to apatite in human dental and skeletal systems (10). The results of studies on the effects of HA have been conflicting, with some studies showing that insertion of hydroxyapatite ingredients into tooth-colored dental materials lead to the improvement of some mechanical properties such as surface hardness (11,12) but some other studies have reported that adding bioactive HA to glass-ionomer cement compromises its properties (13).

In addition, there is some controversy over results of researches designed to investigate the influence of bleaching agents on surface microhardness of restorative materials. The results have shown increase (14-16), decrease (17,18) and no change (19) in surface hardness.

Considering the inconsistency in these outcomes and according to our knowledge; lack of any report on the effect of home bleaching agents on surface hardness of different kind of GICs mixed with HA, the aim of this study was to evaluate the effect of 15% carbamide peroxide gel on the surface hardness of two types of commercial GICs containing 20 %wt. of HA.

## Material and Methods

In this experimental study two different GICs were used: resin-modified glass-ionomer (RMGI) (GC, Gold label, Japan) and Zirco-nomer (Zr) (Shofu INC, Japan). The specimens of each material were divided into 4 groups (n=10): control group, 20 %wt. hydroxyapatite-containing group (HAC), bleached group and bleached 20 %wt. hydroxyapatite-containing group (BHAC).  Table 1 presents the details of restorative materials, their compositions and manufacturers. Bleached group specimens were treated with 15% carbamide peroxide home bleaching agent (Opalescence, Ultradent, South Jordan, USA) for 5 hours a day in a period of 14 days while the specimens in the control groups and HA-containing groups were stored in distilled water for 14 days at room temperature.

**Table 1:**
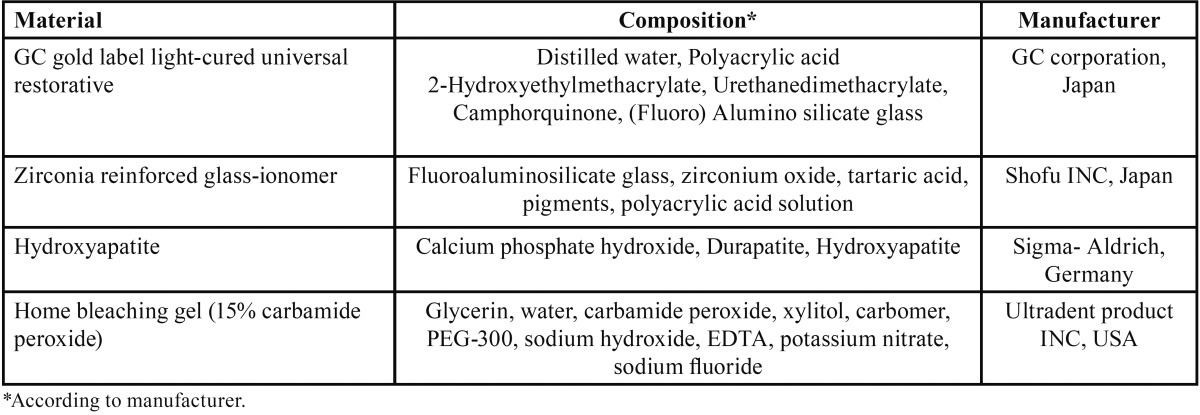
The materials tested.

-Specimen preparation

A disk-shaped plastic mold, 6 mm in diameter and 2 mm in height, was prepared to fabricate the specimens. RMGI powder was mixed with its liquid according to the manufacturer’s proportional recommendation (a powder/liquid ratio of 3.2:1 gr) on a mixing pad with a plastic spatula; then the plastic molds were positioned on the mylar strips lying on the glass plate. The mold was overfilled with the material, covered with mylar strips and pressed flat to extrude excess material. Both sides of the samples were light-cured (20,21) for 20 seconds using a light-emitting diode (LED) polymerizing unit (Monitex, Blue Lex, GT1200, Taiwan) at a light intensity of 1200 Mw/cm2 and 470 nm wavelength. The LED curing probe, 0.8 cm in diameter, was placed exactly in touch with the surface of each specimen.

In order to prepare Zirconomer disks the powder and liquid were mixed at a ratio of 8.0:1.0 g for 30 seconds and plastic molds were filled as discussed above and then left undisturbed for 3 minutes.

To prepare 20% wt. HA-containing GICs, micro-hydroxyapatite powder (Sigma Aldrich, Germany) was added to Zr and RMGI powder at a ratio of 20:80 by weight (22) . Before each specimen preparation, accurate powder ratios of HA/RMGI and HA/Zr were measured by a digital weighing machine (A&D, Japan, 0.0001 accuracy) and then mixed in an amalgamator (Faghihi, FD-4300, Iran) for 20 seconds to produce a homogenous powder. The liquids of RMGI (Fuji II LC) and Zr (Shofu) were used for preparing HA-containing GICs without any modification. HA-containing RMGI disks were light-cured in the same manner as RMGI specimens and the process was carried out as previously discussed.

Following removal of mylar strips both sides of all the specimens were coated with varnish (Kimia, Iran). Then the specimens were stored in distilled water at room temperature for 24 hours. Subsequently, the specimens were polished with the use of a low-speed handpiece with three different disks: medium for finishing, fine for polishing and superfine for super-polishing (Super Snap, Rainbow Technique Kit, Shofu, Japan). In order to minimize inter-operator errors one operator polished all the specimens in one direction with 20 strokes per disk. Finally, the polished disks were cleaned under running distilled water for 1 minute to remove any debris.

In bleached groups 15% carbamide peroxide was applied on the top surface of each specimen with a microbrush and left at room temperature for 5 hours a day for 14 days according to manufacturer’s instructions. After each application of bleaching agent, the specimens were rinsed for 1 minute under running distilled water and then stored again in distilled water at room temperature until the next application. The distilled water used for storage was renewed every day.

-Surface microhardness testing 

To measure surface microhardness (VHN), each sample’s top surface was indented at 3 points by a microhardness testing machine (SCTMC, 1000 Z, China) by using a 300-gr load with a dwell time of 15 seconds. Each indention point was at least 1 mm away from each other or disk border. Then mean of these 3 points was calculated and reported as the surface hardness of each sample.

-Statistical analysis

Statistical analysis of data was performed using SPSS 18.0 (SPSS Inc., Chicago, IL., USA). Three-way ANOVA was used to assess interaction effects. Tukey HSD test was applied to compare the means at a significance level of *p*<0.05.

## Results

 Table 2 presents mean Vickers surface hardness values and standard deviations of tested restorative materials after treatments (bleaching and adding HA). There were significant interactions between GIs and bleaching (F=6.491, *P*=0.013), between GIs and hydroxyapatite (F=45.837, *P*<0.001) and between hydroxyapatite and bleaching (F=113.143, *P*<0.001).

**Table 2:**
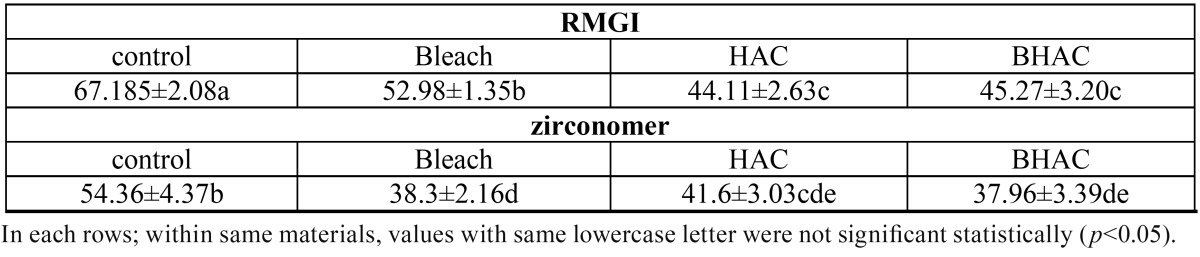
Mean surface microhardness value (VHN) and standard deviations (khf/mm2) in each subgroup.

In this study RMGI had the hardest surface among all the groups. For each material, the control group was significantly harder than the other three groups. With the use of both materials the bleached groups exhibited lower surface hardness than respective non-bleached groups except for the HA-containing RMGI. HA-containing groups exhibited a significant decrease in surface hardness compared to their respective groups with no HA in both tested materials; this reduction was not significant for Zr in the BHAC group compared to its Bleach group (Mean surface microhardness value (VHN) and standard deviations (khf/mm2) in each subgroup.=1.000). the sequence of surface hardness by Zirconomer for treatment groups was as follow: HAC>Bleach >BHAC, but statistical analysis did not reveal any significant difference between them. With regard to RMGI the least value was observed in the HAC and BHAC groups; but the mean surface hardness values was not significantly different (Mean surface microhardness value (VHN) and standard deviations (khf/mm2) in each subgroup.=1.000). According to the Tukey test for Zirconomer in the Bleach group the surface microhardness decreased more in comparison to the HAC group although the difference was not significant (Mean surface microhardness value (VHN) and standard deviations (khf/mm2) in each subgroup.=0.379). In contrast, more reduction was observed in the HAC group compared to the Bleach group for RMGI and the difference was significant (Mean surface microhardness value (VHN) and standard deviations (khf/mm2) in each subgroup.<0.001).

## Discussion

In the present study RMGI was harder than Zr, consistent with the results of a study by Li (23), who showed that RMGI had a higher VHN than conventional GICs but in contrast with the results reported by Xie (24), who measured the Knoop hardness number instead of VHN and showed that conventional glass-ionomers were harder than RMGIs after 7 days of storage in distilled water. The lower surface hardness of Zirconomer-reinforced glass-ionomer might be explained by heterogeneous phases in this cement but SEM photomicrographs are needed for better explanation of the relationship between VHN value and GICs structure.

In this survey we used 15% CP for 5 hours a day for 2 weeks. Based on the results of this study, home bleaching agents decreased surface microhardness of GICs except for HA-containing RMGI whose surface hardness increased but it was not significant (*P*=1.000). The results on the effect of bleaching agent on GIs surface hardness are in agreement with the findings of Malektaher, who showed that surface hardness of RMGI decreased after application of 15% CP (17). In addition, Hao *et al.* reported that surface hardness of conventional GI decreased after the use of 10% CP (18). However, in another study by Hao conflicting results was shown; 15% CP increased surface microhardness of GI cement (16). Some other studies have shown no significant changes in surface microhardness values of glass-ionomer cements (19). These variations can be explained by different brands of GIs used in each study, different percentages of CP applied and differences in experimental methodologies (16). The mechanism of surface softening due to bleaching agents are not still recognized exactly but we know CP degrades into hydrogen peroxide (HP) (1/3) and urea (2/3) (16); HP breaks down into perhydroxyl free radical, which has an oxidizing potential and extensive diffusion ability. Peroxide induces oxidative cleavage of polymer chains and free radicals affect pigment macromolecules as well as resin‒filler interface, resulting in debonding. As long as unreacted double bonds are assumed to be most vulnerable parts of the polymer, a reduction in surface microhardness due to polymer chain cleavage and organic matrix erosion is the result (14,25).

**Figure 1:**
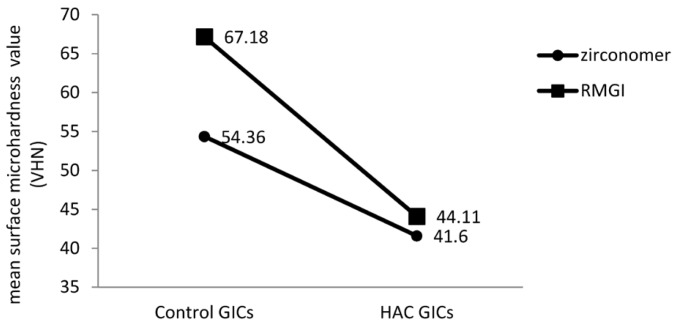
The effect of adding 20% wt. hydroxyapatite (HA) on surface hardness of GICs.

In this study we selected micro-HA since Raul *et al.* reached this idea that when microscopic instead of nanoscopic HA was used as reinforcing filler, mechanical properties such as surface hardness were more favorable (11). We observed a significant reduction in material surface hardness, especially in RMGI after adding 20 %wt. of HA (Fig. 1). Gu *et al.* (12) showed that a composition of 4 an 12 %vol. of hydroxyapatite/zirconium oxide and GICs (HA/ZrO2/GICs) yielded superior mechanical properties com-pared to the original GICs. In addition, they concluded that mechanical properties of HA/ZrO2/GICs were much better than those of HA/GICs. When the amount of HA increased to 28 and 40 %vol. in that study, there was a decrease in surface microhardness. Goenka observed that mean surface hardness value of GIC decreased when the amount of crystalline calcium-deficient hydroxyapatite (CDHA) increased from 5 to 15 %wt. (13). Our results were consistent with some studies that reported adding bioactive glass into GIC powder decreased surface hardness of materials (20,26) but in another study it was indicated that adding nano-HA into conventional glass-ionomer cement promoted mechanical properties (7). Also another study showed that incorporation of 50 to 60 %wt. of HA into the visible-light-cured composite resin can increase surface hardness (11). These inconsistencies in the results of different studies might be attributed to differences in restorative materials used, the percentages and sizes of HA particles and whether HA was added as weight percentage or volume percentage. One reason for the decrease in VHN value when HA was added to RMGI and Zr, might be due to the decrease in the density of set cement after adding HA which contains calcium ions. These ions are more reactive than aluminum cations with carboxylate groups in polyacrylic acid, resulting in less crosslinking between aluminum and carboxylate, weakening the structure (27). When samples are subjected to Vickers surface hardness testing machine after 14 days of immersion in water, HA particles can be dissolved gradually, leading to HA/glass-ionomer degradation and formation of voids, negatively affecting the surface hardness of materials. Furthermore, HA/ZrO2 particle sizes are smaller than those of the glass powder and the surface area is much larger compared to glass; therefore, a greater amount of liquid is necessary for interaction. Since we exactly follow the manufacturer’s instructions and do not change anything, the liquid might be insufficient for dissolving HA/ZrO2 powder completely and the reaction between the powder and liquid would be incomplete. All these processes can compromise the mechanical strength and surface hardness of materials (12).

**Figure 2:**
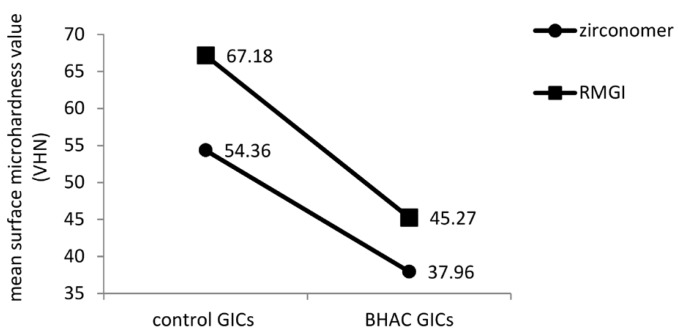
The simultaneous effect of bleaching and adding hydroxyapatite on surface hardness of GICs.

One explanation for the finding that showed adding HA and applying a bleaching agent decreased VHN value in Zr slightly less than that in RMGI (Fig. 2) might be due to the fact that combining ZrO2 with small particle size and glass with large particle size results in wide distribution of particles, leading to high packing density of this cement. In addition, Zr does not dissolve in water by increasing the soaking time. The maturation of RMGI is time-dependent and we applied bleaching only 24 hours after mixing the powder and liquid; therefore, the bleaching agent was used during its maturation. The immature structure is probably more susceptible to degradation, unlike Zr which is resistant to dissolution as soon as it sets completely (12). Therefore, these phenomena can explain why surface hardness reduction is lower in Zr.

According to table 2 one interesting result of this survey was that when a bleaching agent was applied on samples which contained HA, the changes in VHN value was not significant compared to the changes of VHN value in samples without HA which treated by bleaching. It seems that HA compensated the negative effect of bleaching. One possible reason for this result can be due to the fact that HA is a basic material and as CP is degraded into HP which is an acidic agent, HA can probably neutralize HP and decrease its amount to degrade resin‒filler bonds and its infiltration into GIs. This is justifiable by the results of Mena-Seranno who reported that calcium-containing bleaching gel diffuses lower amounts of HP to travel to the pulp chamber compared to the calcium-free bleaching agent due to the reaction of HP surplus with calcium gluconate in its composition and formation of calcium hydroxide, reducing further surplus of HP (28).

According to  Table 2, comparison of the data of samples receiving both HA and the bleaching agent with those of samples containing HA only for each material showed no significant differences in surface hardness values. It seems that adding HA is the main factor responsible for a decrease in surface hardness of HA-containing GICs exposed to the bleaching agent. The possible reason for this finding can be related to the mechanism of HA in decreasing surface hardness, explained previously. Therefore, the materials were influenced by HA, resulting in a decrease in surface hardness. When the bleaching agent was applied, it is possible that HA neutralizes the acidity of HP derived from CP by its alkalinizing potential; therefore, bleaching couldn’t decrease surface hardness more significantly in HA-containing materials.

In vitro studies have some limitations; they cannot simulate clinical conditions properly. In this study, the bleaching agent and materials were not in contact with saliva and debris that can buffer the bleaching agent. Further studies are needed to simulate the oral cavity conditions and different percentages of HA that might change the results. According to the results of this study, it seems that Zr and RMGI might need to be replaced after the bleaching procedure. It should be pointed out that surface hardness is only one of the main factors in evaluation of restorative materials so further studies are necessary to evaluate other mechanical properties.
